# Patients-specific virtual surgical navigation for lung segmentectomy: a prospective multicenter study

**DOI:** 10.3389/fonc.2026.1774607

**Published:** 2026-03-11

**Authors:** Chang Young Lee, Woo Sik Yu, Young Ho Yang, Go Eun Byun, Joonho Jung, Seokjin Haam

**Affiliations:** 1 Department of Thoracic and Cardiovascular Surgery, Severance Hospital, Yonsei University College of Medicine, Seoul, Republic of Korea; 2 Department of Thoracic and Cardiovascular Surgery, Ajou University School of Medicine, Suwon, Republic of Korea

**Keywords:** non-small cell lung cancer, preoperative planning, segmentectomy, surgeon workload, three-dimensional simulation

## Abstract

**Background:**

Anatomical segmentectomy has become a standard surgical option for small peripheral non-small cell lung cancer (NSCLC); however, its technical complexity necessitates precise preoperative planning. This study evaluated the feasibility and clinical utility of the lung module of a patient-specific virtual surgical navigation system for preoperative planning in anatomical segmentectomy and subsegmentectomy.

**Methods:**

This prospective multicenter observational study enrolled 34 patients undergoing anatomical segmentectomy or subsegmentectomy between May and July 2025. Preoperative planning was sequentially performed using conventional two-dimensional (2D) CT and a patient-specific virtual surgical navigation system based on AI-driven three-dimensional (3D) reconstruction. Feasibility outcomes included turnaround time, operational stability, and accuracy of tumor localization, segment prediction, and bronchovascular anatomy compared with intraoperative findings. Surgeon workload was assessed using the NASA Task Load Index (NASA-TLX). Perioperative outcomes were compared with historical cohorts planned using conventional 2D CT and commercially available 3D CT systems after propensity score matching.

**Results:**

All cases achieved successful 3D reconstruction within 72 hours, with complete operational stability. The navigation system demonstrated near-perfect concordance with operative findings for tumor localization and segment prediction (κ = 0.96–1.00), and significantly higher accuracy in predicting resected arteries, veins, and bronchi compared with 2D CT planning. Surgeon workload was significantly reduced with navigation system–based planning (overall NASA-TLX score: 52.5 ± 12.1 vs. 76.1 ± 15.1; p < 0.001), particularly in mental, physical, and temporal demand domains. Compared with both 2D CT and conventional 3D CT planning cohorts, use of the navigation system was associated with shorter operative time, reduced blood loss, and fewer resected subsegments, while maintaining comparable surgical margins and postoperative outcomes.

**Conclusion:**

The lung module of a patient-specific virtual surgical navigation system is a feasible and effective tool for preoperative planning in anatomical segmentectomy and subsegmentectomy. It improves anatomical prediction accuracy, reduces surgeon workload, and demonstrates favorable perioperative performance, supporting its clinical value in technically demanding lung-sparing surgery for early-stage NSCLC.

## 1 Introduction

Lung cancer remains the leading cause of cancer-related mortality worldwide (1). For small (≤2 cm) peripheral non-small cell lung cancer (NSCLC), the JCOG0802/WJOG4607L (2) and CALGB 140503 (3) trials demonstrated non-inferior survival and superior postoperative outcomes after segmentectomy compared with lobectomy, establishing anatomical segmentectomy as a new standard of care. However, the technically demanding nature of segmentectomy—owing to complex anatomical variations and the need for sufficient resection margins—necessitates precise preoperative planning, for which three-dimensional (3D) simulation has become indispensable to ensure anatomical accuracy and safe resection (4, 5). 3D computed tomography (CT) simulation has become integral to surgical planning, enabling visualization of the bronchovascular anatomy and intersegmental planes. Commercially available 3D image analysis systems have improved operative safety but are limited by their high cost, the requirement for dedicated in-hospital software installation, and the extra effort required for surgeons to perform 3D reconstruction themselves (6).

Recently, a patient-specific preoperative virtual surgical navigation system (RUS™, Hutom, Seoul, Korea), which is an AI-based platform, has demonstrated clinical feasibility in other surgical fields. Park et al. (7) reported its usefulness in gastric cancer surgery for accurate vascular mapping and intraoperative guidance, while Lee et al. (8) validated its performance during robotic partial nephrectomy, showing precise anatomical delineation and workflow efficiency. Building upon these findings, the lung module was developed to assist preoperative planning for thoracic segmentectomy and subsegmentectomy, aiming to provide accurate 3D anatomical visualization and reduce surgeon workload.

This study aimed to evaluate the feasibility of the lung module of a patient-specific virtual surgical navigation system in preoperative planning for anatomical segmentectomy or subsegmentectomy. The study also assessed the accuracy of anatomical prediction and resection-margin estimation compared with conventional CT-based planning and compared perioperative outcomes with conventional two-dimensional (2D) and currently available 3D image analysis tools. Additionally, surgeon workload was assessed using the NASA Task Load Index (NASA-TLX) (9).

## 2 Materials and methods

### 2.1 Study design and patient selection

This was a prospective multicenter observational study conducted at two tertiary institutions between May 2025 and July 2025. Thirty-six patients with primary lung cancer scheduled for anatomical segmentectomy or subsegmentectomy were initially enrolled; two were excluded due to dropout, resulting in 34 patients for the final analysis. Eligibility criteria required a preoperative chest CT suitable for 3D reconstruction. Exclusion criteria were prior lung surgery, contraindications to contrast-enhanced CT, or inadequate image quality which precluded reliable anatomical reconstruction (e.g., motion artifacts or insufficient visualization of bronchovascular structures). All participants provided written informed consent, and the protocol was approved by the institutional review boards of both institutions (approval no. 1-2024-0073), in accordance with the Declaration of Helsinki.

### 2.2 CT acquisition and 3D reconstruction

**Figure 1:**
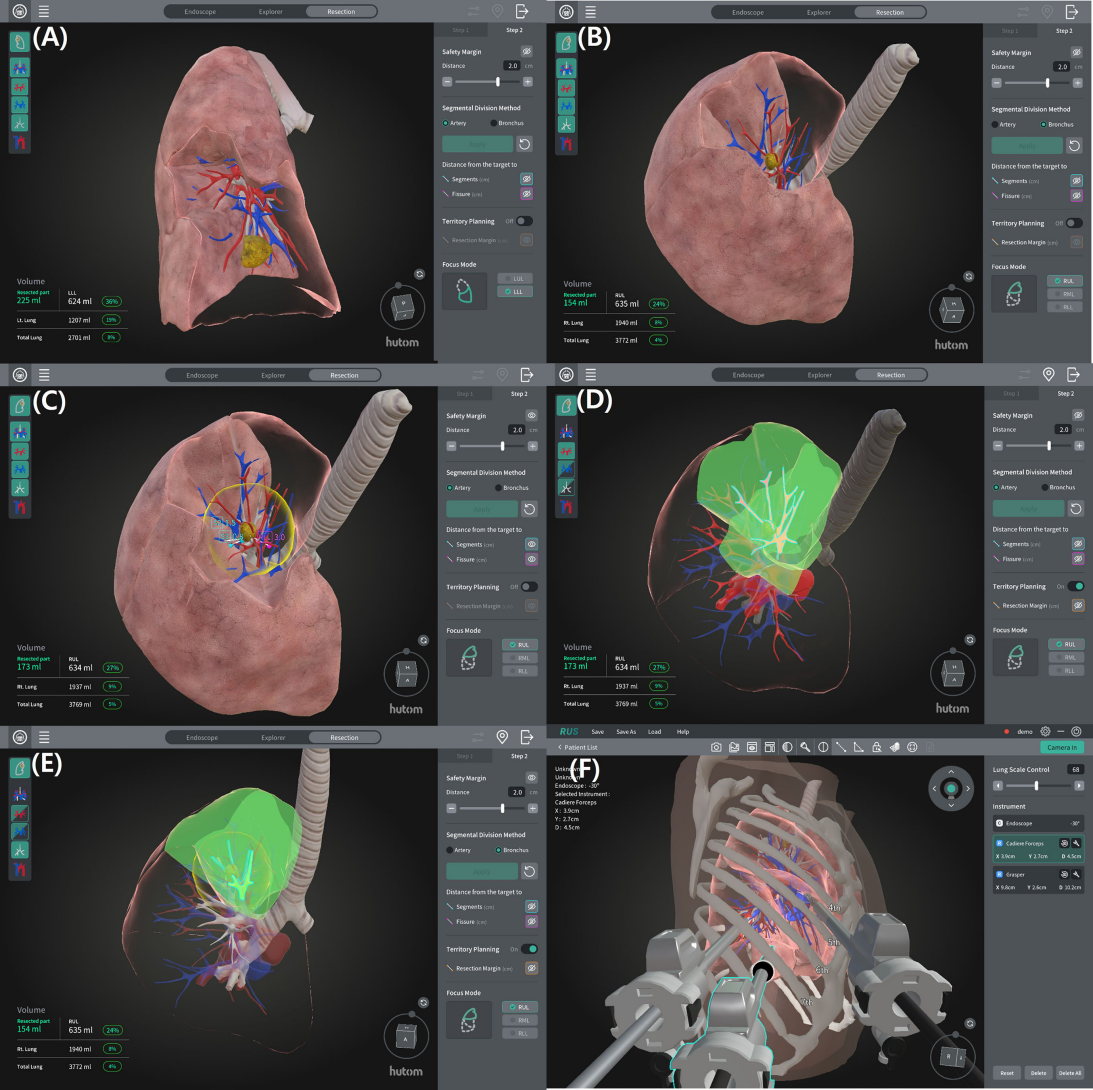
Segmentectomy simulation using navigation system. **(A, B)** Segmental territories are visualized based on artery and bronchus, respectively. **(C)** An adjustable safety margin, along with the distances from the tumor to adjacent segments and the fissure are displayed. **(D, E)** Target segmental arteries and bronchi planned for resection are identified. **(F)** Virtual port placement is simulated based on patient-specific anatomy.

All patients underwent preoperative contrast-enhanced thin-slice CT (1 mm). Digital Imaging and Communications in Medicine (DICOM) files were uploaded to the cloud system through a server. Based on a finely tuned 3D U-Net, artificial intelligence–based segmentation algorithms reconstructed patient-specific 3D models, including the segmental and subsegmental bronchi, pulmonary arteries, veins, and the target lesion. 3D U-Net is a specialized deep learning algorithm for biomedical image segmentation that used its own fine-tuned model trained on clinical data annotated by radiologists. A thoracic surgeon and a radiologist jointly reviewed the reconstructed models for anatomical plausibility. The surgical navigation system additionally enabled virtual resection, estimation of intersegmental planes, measurement of resection margins, and prediction of the number of arteries, veins, and bronchi to be divided (**Figure 1**).

### 2.3 Preoperative planning and simulation

Preoperative planning was performed by four board-certified thoracic surgeons from the participating institutions. All surgeons had prior clinical experience in anatomical segmentectomy. Two surgeons had prior experience with conventional three-dimensional (3D) reconstruction–based preoperative planning, whereas the remaining two surgeons, despite routine experience in segmentectomy, had limited prior exposure to 3D reconstruction software before study initiation. Before patient enrollment, all surgeons completed standardized orientation and training sessions for the navigation system. For each patient, the responsible thoracic surgeon sequentially performed planning with both methods. First, conventional CT-based two-dimensional (2D) planning was carried out using axial, coronal, and sagittal images. Immediately thereafter, the same surgeon performed planning using the surgical navigation system based on reconstructed 3D models. This within-patient sequential design enabled direct comparison of tumor localization, selection of resected subsegments, identification of bronchovascular structures, and resection-margin assessment. The NASA-TLX questionnaire, which evaluates six domains: mental demand, physical demand, temporal demand, performance, effort, and frustration, was completed immediately after each planning session to minimize recall bias. All surgeons received standardized instructions prior to study initiation.

### 2.4 Surgical procedure

All 34 patients underwent anatomical segmentectomy via video-assisted thoracoscopic surgery (VATS) or robotic-assisted thoracic surgery. Extent of resection (simple versus complex segmentectomy and number of resected subsegments) and intraoperative findings were prospectively recorded. Simple segmentectomy was defined as resection involving a single intersegmental boundary, such as left or right S6 segmentectomy (LS6 or RS6), whereas all other procedures, including resections involving multiple segments or subsegments and complex intersegmental planes, were classified as complex segmentectomy. Intersegmental planes were divided primarily using stapling devices, with selective use of energy devices or electrocautery according to anatomical and technical considerations.

### 2.5 Study endpoints

**Figure 2:**
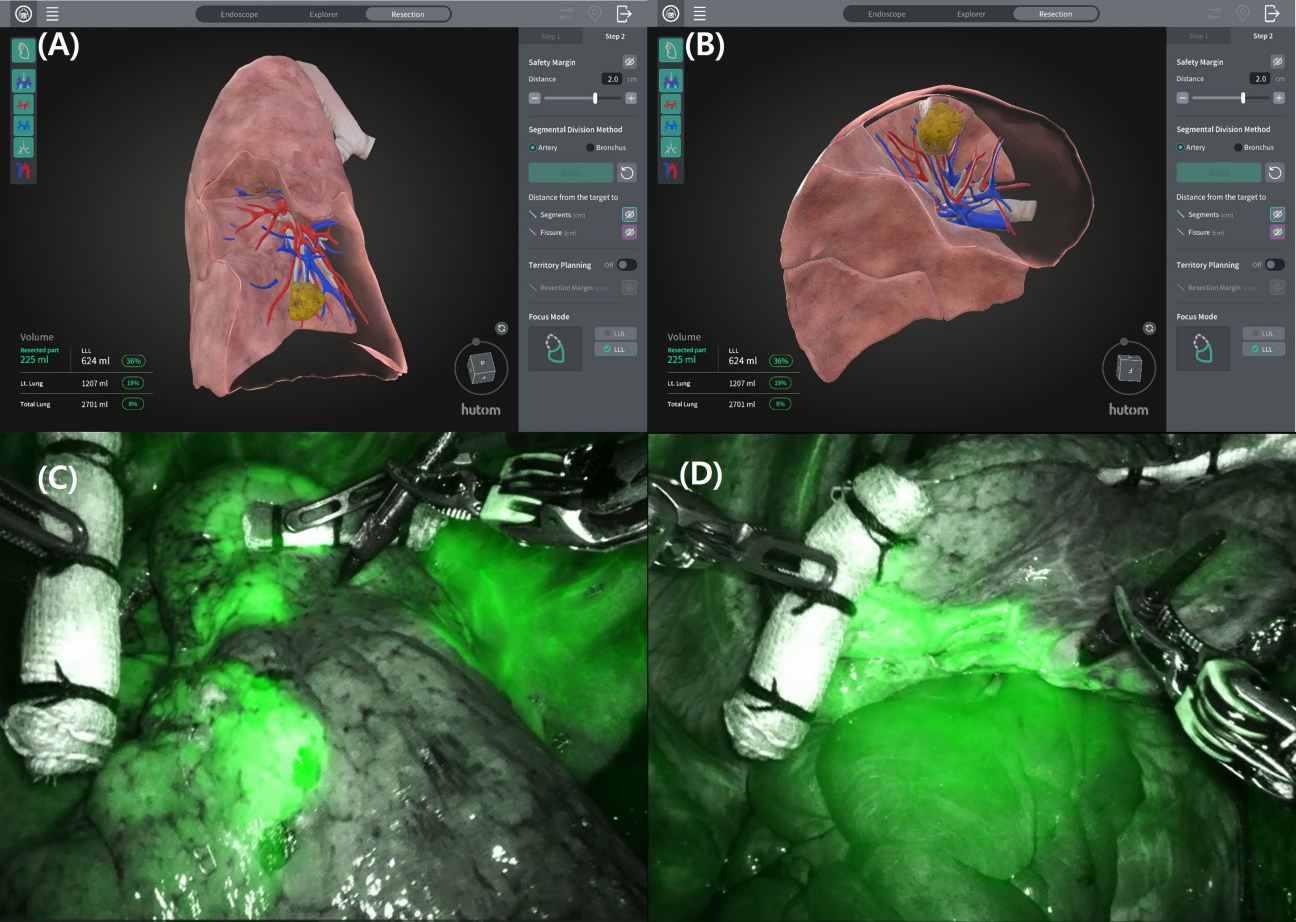
Comparison between the segmental boundaries generated by navigation system and those visualized after intraoperative ICG injection in the left 10 segmentectomy. **(A, B)** 3D segmental territory displayed by navigation system. **(C, D)** Segmental boundaries identified intraoperatively after ICG injection.

The primary endpoint was feasibility. To validate feasibility, the following were evaluated: (1) turnaround time defined as the elapsed time from successful upload of CT DICOM data to completion of the reconstructed 3D model available for surgical planning (<72 hours), (2) operational stability defined as system performance during clinical use and assessed using a composite score evaluating system accessibility, reconstruction completeness, and absence of software-related workflow interruption (0–15 scale), (3) accuracy of tumor localization and segment prediction (Cohen’s κ versus operative findings), (4) prediction accuracy of resected arteries, veins, and bronchi (Cohen’s κ versus operative findings), and (5) resection-margin ratio prediction (difference between planned and pathological margin by calculating the absolute difference between preoperatively predicted margins and pathological margins) (6). The concordance between the preoperatively planned intersegmental plane and the intersegmental plane identified intraoperatively after indocyanine green (ICG) injection (**Figure 2**).

Secondary endpoints evaluated perioperative performance compared with historical cohorts: (i) conventional 2D CT–based planning and (ii) commercially available 3D CT–based planning. Endpoints included operative time, estimated blood loss, number of resected subsegments, margin status, postoperative complications (Clavien–Dindo), and hospital stay. To adjust for potential confounders, propensity score matching (PSM) was performed in a 1:3 ratio using the nearest neighbor matching algorithm with a caliper of 0.2. Matching variables included sex, age, total tumor size, tumor location, segmentectomy type, smoking history, and FEV_1_ (%). After matching, standardized mean differences (SMDs) were calculated to assess intergroup balance, and all SMD values were less than 0.1, indicating good balance between the groups.

### 2.6 Statistical analysis

Continuous variables were summarized as mean ± standard deviation or median with interquartile ranges and compared using the Mann–Whitney U test. Categorical variables were expressed as counts and percentages and compared using Fisher’s exact test. Agreement between planning and operative findings was evaluated with Cohen’s κ. All statistical tests were two-sided, with p < 0.05 considered statistically significant. Analyses were conducted using R software (version 4.3.0; The R Foundation for Statistical Computing, Vienna, Austria).

## 3 Results

### 3.1 Patient characteristics

A total of 36 patients were initially enrolled, of whom 2 were excluded due to lobectomy. The final cohort consisted of 34 patients who underwent anatomical segmentectomy. **Table 1** summarizes the baseline characteristics.

**Table 1:** Baseline characteristics.

Variables	Value
Age (years)	59.9 ± 12.9
Sex (Male/Female)	12 (35.3)/22 (64.7)
Tumor location	
RUL	12 (35.3)
RLL	8 (23.5)
LUL	10 (29.4)
LLL	4 (11.8)
Tumor size (cm)	1.50 ± 0.47
Segmentectomy type	
Simple/Complex	6 (17.6)/28 (82.4)
Subsegmentectomy (n)	11 (32.35)
Resected subsegment (n)	2.56 ± 0.93
Resection margin (cm)	1.80 ± 0.61
Estimated blood loss (ml)	22.4 ± 70.5
Operation time (min)	105.4 ± 43.4
Hospital stay (days)	3.5 ± 1.40
Complications (n)	1 (2.94)

Values are presented as mean ± standard deviation (SD) or number (%). RUL, right upper lobe; RLL, right lower lobe; LUL, left upper lobe; LLL, left lower lobe.

The median age of the patients was 63.0 years (IQR, 55.0–69.5). There were 12 males and 22 females. The median tumor size was 1.50 ± 0.47 cm. The majority of tumors were located in the upper lobes (right upper lobe, n = 12; left upper lobe, n = 10), followed by the right lower lobe (n = 8) and the left lower lobe (n = 4). The median surgical margin was 1.80 ± 0.61 cm, and all margins were pathologically negative.

**Figure 3:**
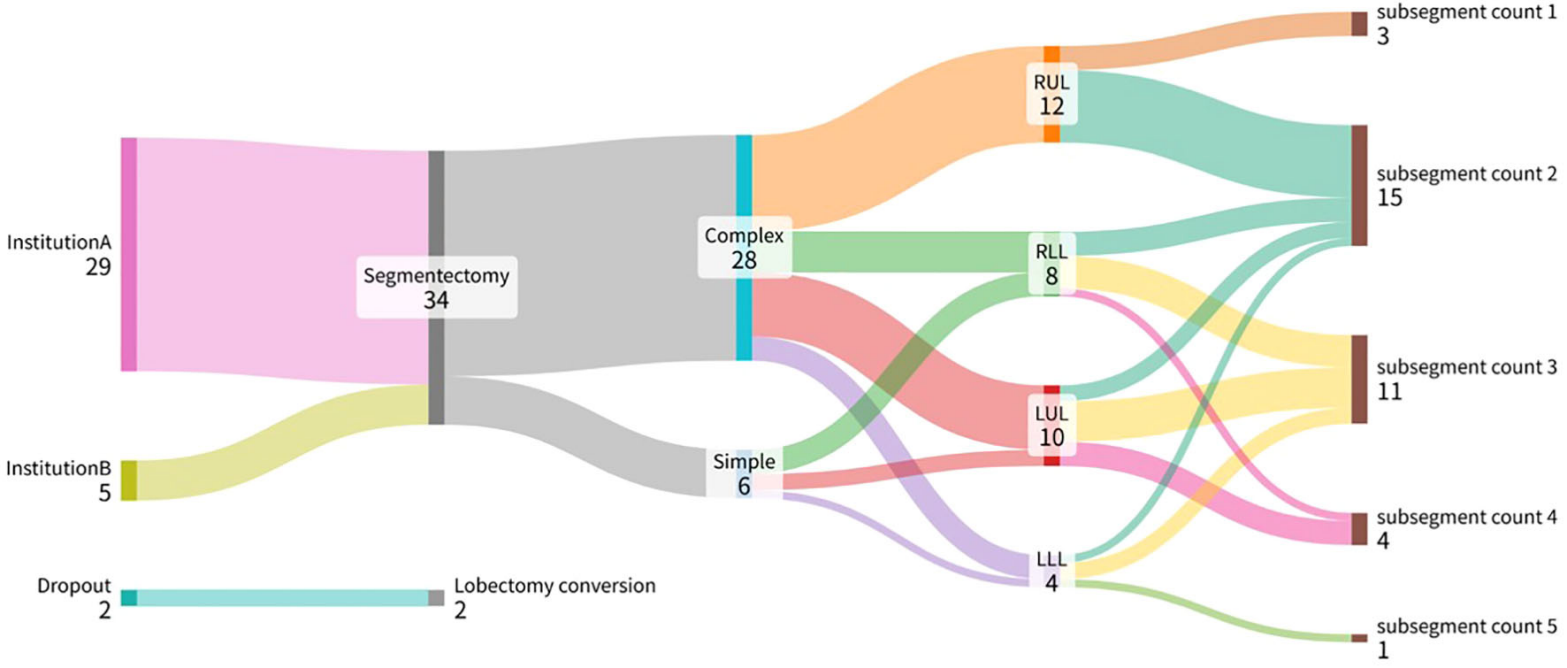
Flow diagram illustrating patient distribution and surgical characteristics.

Procedural distribution is shown in **Figure 3**. Among the 34 resections, 28 were complex and 6 were simple segmentectomies. The most frequent extent of resection involved two subsegments (n = 15), followed by three (n = 11), four (n = 4), one (n = 3), and five (n = 1).

### 3.2 Feasibility

In all patients (34/34), CT images were converted within 72 hours, confirming the timely feasibility of image processing. The median reconstruction turnaround time was 17.92 hours (IQR, 17.22–18.40). The patient-specific virtual surgical navigation system demonstrated complete operational stability with a mean stability score of 15/15 across all patients. Accuracy of segment prediction was 100% (34/34, κ=1.0), indicating perfect concordance between navigation system-based planning and the actual resected segments where the tumor was located.

As summarized in **Table 2**, navigation system-based planning demonstrated consistently higher concordance with intraoperative findings compared to CT-based planning. For tumor location and resected subsegment, navigation system achieved near-perfect agreement (97.1–100%) with κ-values above 0.96, whereas CT showed lower agreement (73.5–85.3%) with κ-values ranging from 0.731 to 0.817. In predicting the number of resected arteries and veins, CT-based planning showed only fair agreement (50.0–61.8%, κ = 0.332–0.390). In contrast, navigation system achieved substantially higher accuracy (91.2–94.1%) with almost perfect concordance (κ = 0.874–0.908). Similarly, for resected bronchi, CT showed limited concordance (κ = 0.248) despite 85.3% agreement, while the navigation system demonstrated 97.1% agreement (κ = 0.920). The agreement score between the RUS Lung reconstruction and the intraoperative ICG-based boundaries demonstrated a mean of 4.62 ± 0.49 on a 5-point scale.

**Table 2:** Agreement between CT-based and navigation system-based planning.

Variables	CT vs. OP			Navigation vs. OP			p-value
	Exact agreement %	Kappa (κ) (95% CI)	N	Exact agreement %	Kappa (κ) (95% CI)	N	
Tumor location subsegment	85.3	0.817 (0.669, 0.965)	34	100.0	1.000 (0.852, 1.000)	34	0.018
Resected subsegment	73.5	0.731 (0.621, 0.840)	34	97.1	0.967 (0.860, 1.000)	34	0.002
No. of resected arteries	50.0	0.332 (0.106, 0.561)	34	94.1	0.874 (0.682, 1.000)	34	< 0.001
No. of resected veins	61.8	0.390 (0.077, 0.702)	34	91.2	0.908 (0.821, 1.000)	34	0.017
No. of resected bronchi	85.3	0.248 (0.026, 0.469)	34	97.1	0.920 (0.811, 1.000)	34	0.001

Values are presented as percentage (%), kappa (κ) value with 95% confidence interval (CI), or number (N). CT, computed tomography; OP, operation; κ, Cohen’s kappa coefficient; CI, confidence interval.

### 3.3 Surgeon’s workload

**Figure 4:**
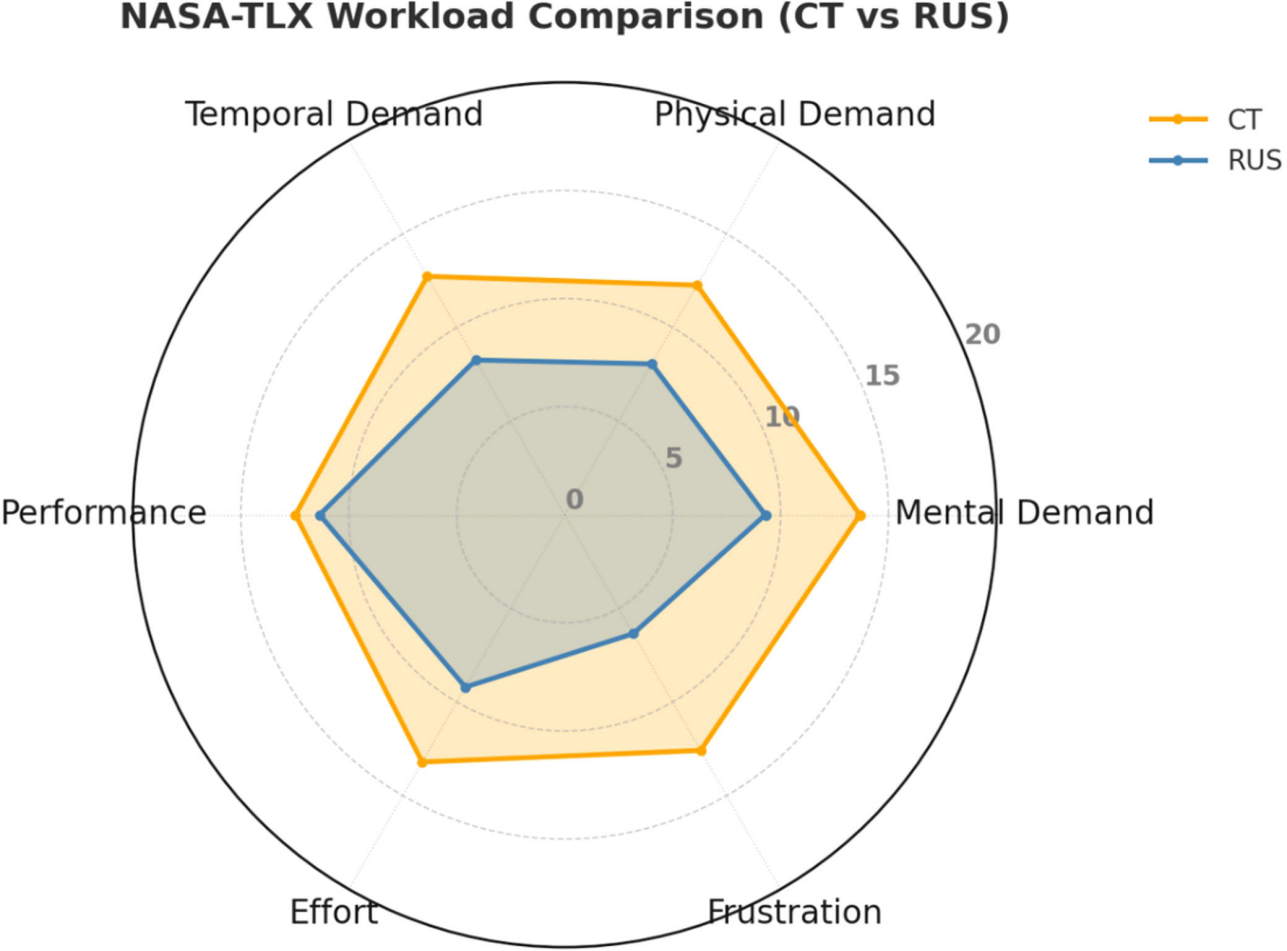
NASA-TLX workload comparison between CT-based and navigation system-based planning.

Assessment of cognitive and physical demands using the NASA-TLX demonstrated significantly lower workload scores with navigation system-based planning compared to CT-based planning (52.53 ± 12.06 vs. 76.06 ± 15.10, *p* < 0.001). As illustrated in **Figure 4**, mental demand was reduced with navigation system (10.6 ± 3.2 vs. 14.1 ± 3.7), as were physical demand (9.3 ± 3.1 vs. 13.0 ± 3.8) and temporal demand (8.1 ± 2.9 vs. 12.5 ± 3.6). Surgeons also reported lower effort (9.5 ± 3.3 vs. 13.4 ± 3.9) and frustration (7.8 ± 2.7 vs. 11.9 ± 3.5) with navigation system-based planning. Performance scores remained similar between groups (10.4 ± 3.0 vs. 10.9 ± 3.2).

### 3.4 Comparisons with historical cohorts

To further evaluate the clinical efficacy of the navigation system, perioperative outcomes were compared with historical cohorts planned using conventional 2D CT (**Table 3**) and 3D CT (**Table 4**). Compared with 2D CT planning, the navigation system was associated with a significantly shorter operative time (96.6 ± 39.6 vs. 122.8 ± 47.8 min, *p* = 0.015), reduced estimated blood loss (99.6 ± 85.9 vs. 174.5 ± 177.4 mL, *p* = 0.001), and a lower number of resected subsegments (2.8 ± 0.8 vs. 3.4 ± 1.2, *p* = 0.012), while maintaining comparable resection margins and complication rates.

**Table 3:** 2D CT vs. Navigation system (N = 102).

Variables	2D CT	Navigation system	p-value
	(N = 73)	(N = 29)	
Age (years)	60.58 ± 10.75	60.31 ± 13.45	0.917
Sex (Male/Female)	28 (38.4)/45 (61.6)	10 (34.5)/19 (65.5)	0.715
Tumor size (cm)	1.59 ± 0.46	1.52 ± 0.45	0.468
Tumor location			0.962
RUL	21 (28.8)	8 (27.6)	
RLL	19 (26.0)	7 (24.1)	
LUL	23 (31.5)	10 (34.5)	
LLL	10 (13.7)	4 (13.8)	
Smoking history			0.499
Never	54 (74.0)	20 (69.0)	
Former	11 (15.1)	7 (24.1)	
Current	8 (11.0)	2 (6.9)	
FEV1 (%)	85.84 ± 15.23	86.93 ± 12.20	0.731
Preoperative localization (n)	3 (4.1)	0 (0.0)	0.556
Segmentectomy type			
Simple/Complex	18 (24.7)/55 (75.3)	6 (20.7)/23 (79.3)	0.670
Subsegmentectomy (n)	15 (20.5)	8 (27.6)	0.443
Resected subsegment (n)	3.44 ± 1.62	2.62 ± 0.90	0.002
Resection margin (mm)	22.21 ± 10.38	18.41 ± 9.02	0.091
Operation time (min)	106.15 ± 47.70	89.59 ± 20.66	0.016
Estimated blood loss (ml)	42.74 ± 64.34	9.66 ± 23.68	< 0.001
Hospital stay (days)	3.82 ± 1.77	3.55 ± 1.50	0.470
Complications (n)	3 (4.1)	1 (3.4)	1.000

Values are presented as mean ± standard deviation (SD) or number (%). 2D CT, two-dimensional computed tomography; RUL, right upper lobe; RLL, right lower lobe; LUL, left upper lobe; LLL, left lower lobe; FEV1, forced expiratory volume in 1 second.

**Table 4:** 3D CT vs. Navigation system (N = 108).

Variables	3D CT	Navigation system	p-value
	(N = 80)	(N = 28)	
Age (years)	62.20 ± 8.791	61.11 ± 12.98	0.682
Sex (Male/Female)	26 (32.5)/54 (67.5)	10 (35.7)/18 (64.3)	0.758
Tumor size (cm)	1.55 ± 0.44	1.55 ± 0.44	0.961
Tumor location			0.967
RUL	24 (30.0)	7 (25.0)	
RLL	19 (23.8)	7 (25.0)	
LUL	26 (32.5)	10 (35.7)	
LLL	11 (13.8)	4 (14.3)	
Smoking history			0.843
Never	57 (71.3)	19 (67.9)	
Former	16 (20.0)	7 (25.0)	
Current	7 (8.8)	2 (7.1)	
FEV1 (%)	87.71 ± 14.88	86.79 ± 12.40	0.768
Preoperative localization (n)	0 (0.0)	0 (0.0)	–
Segmentectomy type			
Simple/Complex	19 (23.8)/61 (76.3)	6 (21.4)/22 (78.6)	0.802
Subsegmentectomy (n)	11 (13.8)	7 (25.0)	0.169
Resected subsegment (n)	3.44 ± 1.22	2.68 ± 0.86	0.001
Resection margin (mm)	22.16 ± 12.71	18.50 ± 9.18	0.111
Operation time (min)	104.08 ± 41.63	90.64 ± 20.23	0.028
Estimated blood loss (ml)	32.63 ± 31.17	10.00 ± 24.04	< 0.001
Hospital stay (days)	4.00 ± 2.34	3.57 ± 1.53	0.369
Complications (n)	5 (6.3)	1 (3.6)	1.000

Values are presented as mean ± standard deviation (SD) or number (%). 3D CT, three-dimensional computed tomography; RUL, right upper lobe; RLL, right lower lobe; LUL, left upper lobe; LLL, left lower lobe; FEV1, forced expiratory volume in 1 second.

When compared with 3D CT planning, navigation system also demonstrated reduced operative time (101.3 ± 40.7 vs. 127.2 ± 48.9 min, *p* = 0.001) and blood loss (111.7 ± 92.5 vs. 180.8 ± 159.7 mL, *p* = 0.001), with fewer resected subsegments (2.7 ± 0.9 vs. 3.4 ± 1.1, *p* = 0.003). Resection margin, complication rates, and hospital stay were comparable between the two groups.

## 4 Discussion

This prospective multicenter study demonstrated the feasibility and efficacy of the lung module of a patient-specific virtual surgical navigation system, a novel surgical navigation system designed for anatomical segmentectomy and subsegmentectomy. These findings indicate that the navigation system offers distinct advantages over conventional 2D CT planning, with significantly shorter preoperative planning times and improved accuracy in tumor localization, anatomical identification, and margin prediction. Importantly, surgeon workload, measured using the NASA-TLX, was consistently lower across all domains except performance, suggesting that the navigation system not only enhances planning efficiency but also alleviates cognitive and physical demands on the surgeon. In addition, when compared with both 2D CT and commercially available 3D image analysis software, the navigation system demonstrated superior or at least comparable performance in perioperative outcomes, showing favorable trends toward reduced operative time and blood loss. Collectively, these results confirm the lung module of a patient-specific virtual surgical navigation system as a feasible, reliable, and clinically efficient tool, particularly in technically demanding procedures such as complex segmentectomy and subsegmentectomy.

Following the results of the JCOG0802/WJOG4607L (2) and CALGB 140503 (3) trials, segmentectomy has been established as a new standard for peripheral non-small cell lung cancer measuring 2 cm or less, demonstrating non-inferiority to lobectomy. Nevertheless, segmentectomy is technically more demanding than lobectomy, as anatomical variations are more frequent and insufficient surgical margins can increase the risk of local recurrence (2, 10). For these reasons, the use of preoperative 3D simulation has been strongly recommended to ensure adequate margin and safe surgical planning (4, 5). Several commercially available 3D simulation platforms have already demonstrated clinical usefulness in preoperative planning for segmentectomy. Among them, Synapse 3D (FUJIFILM, Japan) and the Visible Patient program (France) are the most widely used, yet their approaches differ substantially. Synapse 3D is an on-premise system integrated with the hospital PACS, allowing rapid reconstruction and real-time surgical planning; however, its adoption is often limited by high initial acquisition costs and the need for local installation and maintenance (6). In contrast, Visible Patient operates as a cloud-based service in which CT data are uploaded externally, and expert-generated 3D reconstructions are returned within 72 hours. While this model eliminates the need for local installation and provides high-quality segmentation, it carries disadvantages such as delayed turnaround time, potential data security concerns, and cumulative costs with frequent use. The surgical navigation system used in this study also provides a service model similar to Visible Patient; however, potential concerns related to turnaround time or operational stability commonly associated with such service-based platforms were not observed in this study.

Consistent with previous studies (10–15) on the 3D simulation systems for segmentectomy, this study indicates that the navigation system provides a more reliable prediction of tumor localization and the resected segment than 2D planning. The superiority of the navigation system was most evident during subsegmentectomy, where accurate delineation of vascular anatomy is particularly critical. Importantly, this observation mirrors prior results obtained with the stomach module of the navigation system (7), suggesting that the system’s advantage in vascular prediction may represent a generalizable strength across different organs.

A distinctive feature of the present study, compared with previous reports on 3D simulation software, is the systematic evaluation of surgeon workload using the NASA-TLX when planning with the navigation system versus conventional 2D CT. This methodology is an established and validated tool for quantifying subjective workload among surgeons. Its application has been shown to reflect both cognitive strain and ergonomic burden during minimally invasive and image-guided procedures (16–19). Specifically, the navigation system reduced mental demand by eliminating the need for surgeons to reconstruct 3D anatomy from multiple 2D images in their minds. It lowered physical demand by minimizing prolonged visual strain and repetitive image review. Temporal demand was reduced through shorter planning time, thereby lessening the time pressure inherent to busy surgical schedules. Although performance scores showed a trend towards improvement, this difference did not reach statistical significance, suggesting that both methods provided comparable levels of perceived task accomplishment and indicating that the reduction in workload did not compromise planning quality. Nevertheless, the system substantially decreased overall effort by simplifying the interpretation of complex vascular and bronchial anatomy with intuitive visualization, and it mitigated frustration by reducing uncertainty and stress associated with anatomical variations. Taken together, these findings indicate that the navigation system not only enhances planning accuracy but also provides comprehensive reductions across most dimensions of surgeon workload.

In clinical practice, most segmentectomies are now performed with the aid of 3D simulation programs, making it difficult to design a randomized study directly comparing 2D planning with 3D navigation. Therefore, we relied on comparisons with existing data rather than prospective randomization. These analyses revealed overall trends toward reduced operative time and decreased estimated blood loss when using the navigation system, while maintaining surgical margin accuracy. Moreover, by reducing the number of subsegments resected, the system maximized the potential for lung function preservation, underscoring a key clinical advantage of this approach.

Despite these promising findings, our study has several limitations. First, the sample size was relatively small, and although conducted prospectively, it was limited to two institutions, which may restrict the generalizability of our results. Second, the comparison with conventional 2D planning and other 3D programs relied partly on retrospective data rather than randomized allocation, which introduces inherent selection bias, and the intent of this comparison was not to suggest inferiority of current commercial systems, but rather to contextualize the performance of the navigation system within existing clinical workflows. Third, while we demonstrated reductions in surgeon workload, the subjective nature of NASA-TLX scoring may not fully capture all aspects of cognitive and technical burden. Fourth, the fixed sequential planning design, in which 2D CT planning always preceded navigation system–based planning, may have introduced order or familiarity effects. Fifth, the operational stability score was developed specifically for this feasibility study and was assessed by the operating surgeon immediately after planning. We acknowledge that this score has not been externally validated. Finally, long-term oncologic outcomes such as local recurrence and overall survival were beyond the scope of this study and require further investigation. Future multicenter randomized studies with larger cohorts and longer follow-up will be essential to validate these findings and to establish the navigation system as a standard tool for segmentectomy and subsegmentectomy planning.

This study demonstrates that the lung module of a patient-specific virtual surgical navigation system is feasible and effective for anatomical segmentectomy and subsegmentectomy. Compared with conventional 2D planning and existing 3D platforms, the navigation system improved planning efficiency, reduced surgeon workload, and maintained surgical margin accuracy, while showing favorable perioperative outcomes and potential benefits in lung function preservation. These results support that the navigation system can serve as a reliable and clinically valuable tool, enabling the safe and efficient adoption of segmentectomy in the management of early-stage non-small cell lung cancer.

## Data Availability

The original contributions presented in the study are included in the article/supplementary material. Further inquiries can be directed to the corresponding author.
